# Dual Polarimetric Radar Vegetation Index for monitoring forest moisture stress using time series of Sentinel‐1 SAR data

**DOI:** 10.1111/plb.70036

**Published:** 2025-05-16

**Authors:** B. Ranjit, W. Bijker, H. Aghababaei, A. Stein

**Affiliations:** ^1^ Department of Earth Observation Science, Faculty of Geo‐Information Science and Earth Observation (ITC) University of Twente Enschede The Netherlands

**Keywords:** Broadleaf deciduous forest, drought stress, forest response, median deviations, radar data

## Abstract

- Global warming and anthropogenic climate change have intensified drought occurrences, raising concerns about their escalating frequency, intensity, and persistence. With the projection that droughts will increase at the end of the century, it is important to find efficient and cost‐effective methods to assess and monitor drought impacts. We leverage freely available satellite‐based remote sensing images to study drought stress in forest.
- In this study, we evaluate the impact of intense and prolonged drought on temperate broadleaf deciduous forests using Sentinel‐1 (S1) Synthetic Aperture Radar (SAR) time series data. For the first time, we used the S1‐derived Dual Polarimetric Radar Vegetation Index (DpRVI) to detect and characterize drought effects in forests. Monthly median DpRVI deviations were obtained from S1 SAR images acquired between 13 October 2014 and 3 July 2023.
- The forest exhibited drought effects through a decline in DpRVI during droughts. These can be attributed to both reduced canopy branches and leaves, and decreased canopy water content. The onset of drought effects in 2018 was captured with negative median DpRVI deviations. An accumulated effect of the multi‐year drought 2018–2020 occurred, as evident by increased negative median DpRVI deviations in the subsequent years up to 2021.
- This study demonstrates the potential of using S1‐derived DpRVI to assess the impacts of droughts on broadleaf forest canopies. Further investigation should be carried out to discriminate the relative contributions of the declining canopy water content and changes in the amount and structure of canopy branches and leaves to the observed DpRVI decline.

## Graphical abstract

This study demonstrates the potential of the Sentinel‐1 Dual Polarimetric Radar Vegetation Index, combined with climate variables and the Standardized Precipitation–Evapotranspiration Index, to effectively detect and monitor drought‐induced stress in temperate broadleaf deciduous forests.

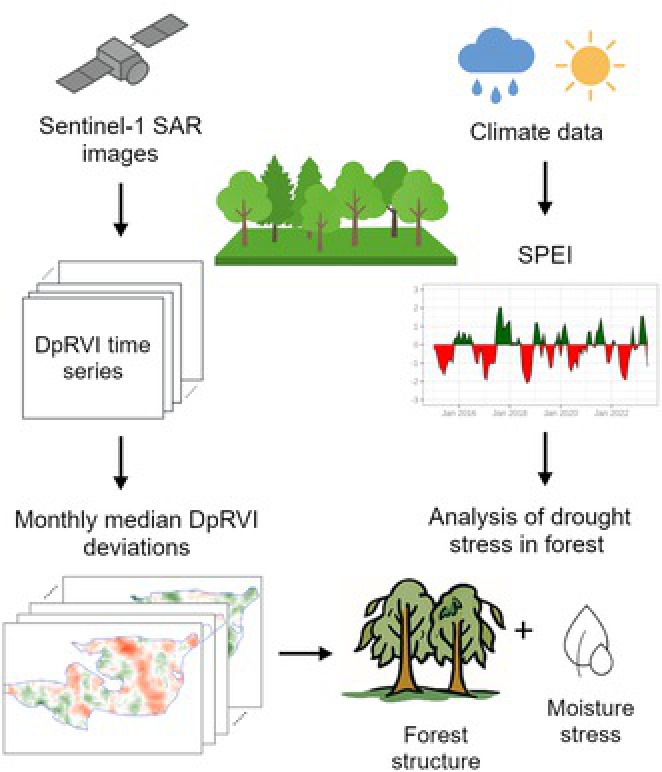

## INTRODUCTION

Anthropogenic climate change and global warming have contributed to exacerbation of drought occurrences. Droughts have not only increased in frequency, intensity, and scale but have also become more persistent, spanning multiple years (Dai 2011; Erfurt *et al*. 2019). The frequency of severe droughts is projected to increase by the end of this century (Orth *et al*. 2016; Konapala *et al*. 2020).

Droughts pose significant threats to forest ecosystems, causing physiological damage, such as water stress, reduced photosynthesis, and hydraulic failure. These stressors lead to canopy damage, including leaf wilting, early senescence, and dieback, which directly impact tree health and function (Hammond *et al*. 2022). Canopy greenness is a key indicator of photosynthetic activity and overall forest health. Droughts result in a decline in canopy greenness in tropical forests (Zhou *et al*. 2014), as well as in temperate forests (West *et al*. 2022). Moreover, drought‐stressed forests become more susceptible to attacks by pests and pathogens, leading to diseases and infections (Anderegg *et al*. 2020). Notably, multiple studies have reported significant declines in canopy moisture content due to droughts across different forest types. For example, Asner *et al*. (2016) observed substantial canopy moisture loss in mixed conifer–oak, pine, and chaparral woodlands in California during the 2012–2015 drought. In the tropics, Frolking *et al*. (2011) detected strong and widespread negative backscatter anomalies indicating canopy moisture loss in southwestern Amazonian forests during the 2005 drought, while Saatchi *et al*. (2013) reported similar declines in Amazonian forests during the 2005 and 2010 droughts. Forest fire incidences are on the rise in response to drought and soaring temperature (World Meteorological Organization 2018). The heightened frequency and severity of droughts have reduced the capacity of forest canopies to retain moisture, making them more vulnerable to fire (Saatchi *et al*. 2013; Yebra *et al*. 2013; Verhegghen *et al*. 2016).

The response of forests to droughts is spatially heterogeneous, with varying degrees of impact both within a forest stand as well as at landscape and regional scale. While droughts often have detrimental effects on most forest ecosystems, some contrasting effects have been observed at higher latitudes. During the summer drought of 2010, forests in Russia (Flach *et al*. 2018) and Scandinavia (Bastos *et al*. 2020) showed increased productivity because of increased solar radiation created more favourable conditions for growth. While some forests exhibit resilience to droughts, others show varied response times. Forest response times to drought events vary widely depending on the severity and duration of the droughts, tree species, soil conditions, and topography (Allen *et al*. 2010). While the time it takes to see the effects of drought varies, so does the time it takes for forests to recover (West *et al*. 2022). Time‐series of remote sensing data is a robust and consistent approach to estimate the spatial and temporal impacts of droughts on forest health, resilience, and recovery by estimating changes in forest structure and moisture content over time.

Time series of satellite imagery can be employed to analyse forest responses to droughts because of their repeated observations across extensive areas which enables non‐invasive monitoring of forest canopies (Verbesselt *et al*. 2010). Optical images have been widely utilized for assessing the impact of droughts on forests and forest recovery thereafter. The Normalized Difference Vegetation Index (NDVI) is commonly used for monitoring forest drought conditions because of its sensitivity to changes in vegetation greenness, leaf area index, and green biomass (Rita *et al*. 2020; West *et al*. 2022). The decline in NDVI during droughts serves as an indicator of drought stress in forests. NDVI anomalies, deviations from normal vegetation conditions, provide information on the severity and spatial pattern of drought impacts on forests (Philipp *et al*. 2021). However, NDVI for drought monitoring has several limitations: it is hindered by cloud cover since it is derived from optical images, and it tends to saturate over areas with dense forest canopies, resulting in a breakdown of the relationship between NDVI and canopy dynamics (Wardlow *et al*. 2012).

The NDVI‐based statistical metrics, such as means and percentiles, have been extensively used in drought impact studies on forests (e.g. Rita *et al*. 2020; Thonfeld *et al*. 2022; West *et al*. 2022). A major drawback of widely employed NDVI‐based statistical metrics is that a considerable amount of data must to be omitted due to cloud cover and shadows (Zhang & Roy 2017). As a result, the number of valid observations available to compute the percentiles varies unpredictably across pixels within the study period, potentially leading to an insufficient valid observations to correctly characterize the forest responses (Xie *et al*. 2020). In a forest drought impact study using Landsat imagery and based on NDVI median deviations, West *et al*. (2022) reported that <50% of pixels were cloud‐free, with no cloud‐free pixels during 21% of the growing season over the 20‐year study period in one‐third of the monthly Landsat composites. These gaps were similarly reported for other optical sensors, such as MODIS and Sentinel‐2, for the same reasons. Irrespective of the optical sensor used, this cloud issue persists.

Synthetic Aperture Radar (SAR) images can overcome the above limitations. Unlike passive optical sensors, SAR sensors are active and operate independently of daylight and weather conditions, ensuring consistent observations regardless of cloud cover. Moreover, SAR signals can penetrate into dense forest canopies, to depths depending on the wavelength (Konings *et al*. 2021). There are still limitations of SAR data. Such data are complex and inherently noisy, making them difficult to interpret compared to optical imagery. SAR data are influenced by factors including topography, soil moisture, forest type, and sensor parameters. Consequently, advanced and challenging processing techniques are required to extract meaningful information for forest monitoring. Recently, availability of longer and denser SAR time series on a global scale proved promising for studying drought impacts. Sentinel‐1 (S1) C‐band SAR images are freely accessible, offering both high spatial and temporal resolution. To reveal drought impacts in forests, however, it is necessary to first analyse the sensitivity of the S1 SAR C‐band time series.

Radar backscatter has been successfully used to study the seasonal and interannual changes in canopy water content and structure (e.g. canopy biomass) (Saatchi *et al*. 2013; Konings *et al*. 2019). Leblon *et al*. (2002) reported a strong correlation between rates of changes in radar backscatter and water content in closed forest canopies of jack pine and spruce, particularly in the high‐frequency C‐band for which the contribution of the underlying soil is negligible. Apart from moisture content, radar backscatter is also sensitive to forest structure (Ulaby *et al*. 1982). Nevertheless, several studies have indicated that S1‐derived indices outperform the direct use of radar backscatter for vegetation studies, as outlined below.

For vegetation studies, several S1 indices have been explored, each with varying sensitivity to moisture content and biomass. These indices are summarized in Table 1. The cross‐pol backscatter ratio $\sigma_{\mathrm{VH}}^{0}/\sigma_{\mathrm{VV}}^{0}$, dual‐pol Radar Vegetation Index formulated $\left({\mathrm{RVI}}_{\mathrm{dp}}\right)$, and Dual Polarimetric Radar Vegetation Index (DpRVI) show a positive correlation with vegetation water content (Kim *et al*. 2012, 2014; Huang *et al*. 2016; Veloso *et al*. 2017; Vreugdenhil *et al*. 2018; Bhogapurapu *et al*. 2021, 2022). Similarly, Dual Polarimetric SAR Vegetation Index (DPSVI) and Polarimetric Radar Vegetation Index (PRVI) have been positively correlated with biomass in terrestrial crops and Mediterranean shrublands (Periasamy 2018; Gururaj *et al*. 2019). Compared to the radar backscatter, RVI has greater sensitivity to canopy moisture changes and reduced sensitivity to variation in structure and environmental conditions, such as soil moisture (Kim *et al*. 2012, 2014; Huang *et al*. 2016). The S1 cross‐pol backscatter ratio $\sigma_{\mathrm{VH}}^{0}/\sigma_{\mathrm{VV}}^{0}$ reduces the double‐bounce effect, specifically the soil–vegetation interaction and soil moisture variation effect (Veloso *et al*. 2017; Vreugdenhil *et al*. 2018). Additionally, compared to individual S1 backscatter coefficients $\sigma_{\mathrm{VH}}^{0}$ and $\sigma_{\mathrm{VV}}^{0}$, the S1 cross‐pol backscatter ratio $\sigma_{\mathrm{VV}}^{0}/\sigma_{\mathrm{VH}}^{0}$ displayed pronounced seasonality in a temperate mixed forest (Frison *et al*. 2018).

**Table 1:** S1 indices used for vegetation studies, including their acronyms, mathematical formulations, and references.

S1 index	formulation	reference
Cross‐pol backscatter ratio	$\sigma_{\mathrm{VH}}^{0}/\sigma_{\mathrm{VV}}^{0}$	Blaes *et al.* (2006)
Dual‐pol Radar Vegetation Index $\left({\mathrm{RVI}}_{\mathrm{dp}}\right)$	${\mathrm{RVI}}_{\mathrm{dp}}={4\sigma }_{\mathrm{VH}}^{0}/\left(\sigma_{\mathrm{VH}}^{0}+\sigma_{\mathrm{VV}}^{0}\right)$	Gururaj *et al*. (2019)
Dual Polarimetric SAR Vegetation Index (DPSVI)	$\text{DPSVI}=\left(\sigma_{\mathrm{VH}}^{0}+\sigma_{\mathrm{VV}}^{0}\right)/\sigma_{\mathrm{VV}}^{0}$	Periasamy (2018)
Polarimetric Radar Vegetation Index (PRVI)	$\text{PRVI}=\left(1-\mathrm{DOP}\right)/\sigma_{\mathrm{VH}}^{0}$	Chang *et al.* (2018)
Dual Polarimetric Radar Vegetation Index (DpRVI)	See Dual Polarimetric Radar Vegetation Index	Mandal *et al*. (2020), Mandal *et al*. (2020)

Here, $\sigma^{0}$ is the backscatter coefficient, with subscripts VH and VV indicating polarization, and DOP refers to degree of polarization.

Among these widely used S1 indices, we chose DpRVI for our study because of its higher sensitivity and improved estimation of biophysical parameters, such as Plant Area Index, Vegetation Water Content and Biomass (Bhogapurapu *et al*. 2021, 2022) in crops, for example, canola, wheat and soybean. Notably, canola, with broadleaf canopies, produced the best performance among these crops. Hence, we anticipate that DpRVI will exhibit better performance, particularly in broadleaf and taller forest canopies, compared to the other indices. Given the structural similarities between broadleaf forests and canola crops, especially in leaf size and dense canopies, we adapted S1 DpRVI for broadleaf forests.

The objective of this study was to assess whether the time series of the S1 DpRVI index enables detection of drought impacts in temperate broadleaf deciduous forest. To our knowledge, this is the first study to apply DpRVI for this. We assessed this in the Rhön Biosphere Reserve, Germany.

## MATERIAL AND METHODS

### Study area

**Fig. 1:**
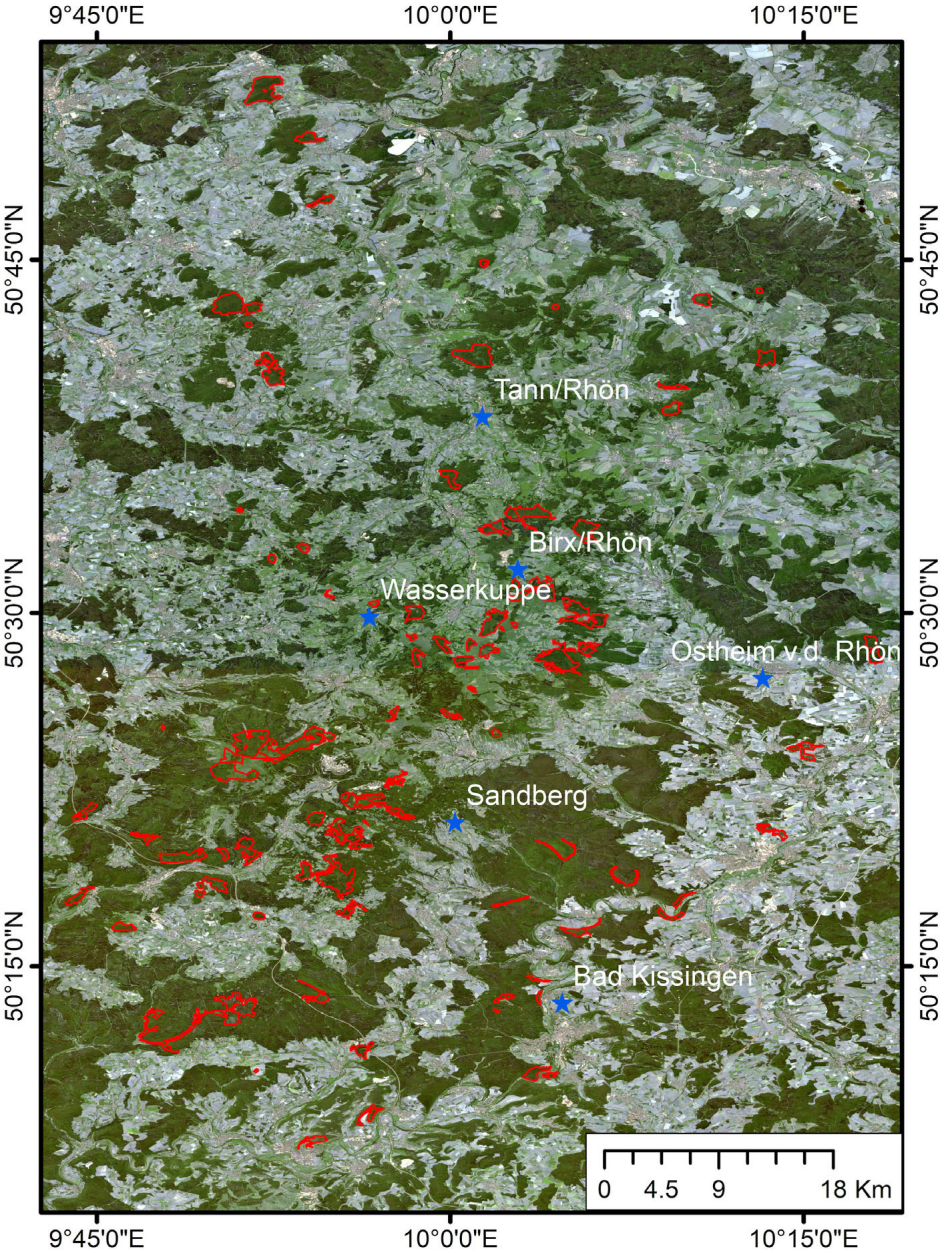
Location of the Rhön Biosphere Reserve plotted over the Sentinel‐2 RGB image of 1 July 2018. The study area is the core forest area polygons shown with red boundaries. The location of the surrounding weather stations is depicted with blue stars.

The Rhön Biosphere Reserve, Germany (Fig. 1), was chosen as our study area because severe droughts have occurred in 2018 and 2019. These droughts impacted European beech (*Fagus sylvatica*) forest, with effects ranging from widespread defoliation, and reduction in leaf chlorophyll, to tree mortality (Schuldt *et al*. 2020). These effects were quantified using NDVI quantiles from MODIS images. The effects of these droughts were widespread but highly heterogeneous. The study area was 72,802 ha, of which 2.3% is designated as core areas (UNESCO 2022) that do not undergo any management intervention. Therefore, the impacted trees were left standing after drought events, allowing assessment of forest recovery and sustained canopy impacts, while in the surrounding production forest, the impacted trees were felled to avoid economic losses. As a result, it is not possible to assess drought impacts in the surrounding production forests. Since the core areas (shown in red boundaries in Fig. 1) are untouched, they are ideal sites to assess drought impacts and were chosen for our study. The average plot size of the core area polygons is ca. 69.60 ha, with plot size ranging from ca. 2.55 ha to 394.57 ha.

### Climate data

The weather data from six weather stations (location in Fig. 1 as blue, stars), namely Tann/Rhön, Birx/Rhön, Wasserkuppe, Sandberg, Ostheim von der Rhön, and Bad Kissingen were downloaded from the German Weather Service for January 1990 to July 2023 (Wetterdienst 2023), as a long baseline period of at least 30 years which captures natural variability is necessary for the calculation of drought indices (Vicente‐Serrano & National Center for Atmospheric Research Staff 2024). These data included daily precipitation (mm), daily temperature (°C), and daily snowfall (cm). The mean annual temperature and precipitation of the study area, calculated from the six weather stations are 8.91°C and 805 mm, respectively. For each weather station, monthly total precipitation was obtained by aggregating daily precipitation values. The monthly means of daily maximum and minimum temperature were obtained by averaging daily maximum and minimum temperatures, respectively.

### Standardized Precipitation Evapotranspiration Index

Standardized Precipitation Evapotranspiration Index (SPEI) is a drought indicator based on climate data used to monitor dry and wet conditions. In this study, SPEI was calculated for the weather stations described above to identify the period when the forest is subject to drought conditions. First, potential evapotranspiration (PET) was calculated using the monthly mean of daily maximum and minimum temperature downloaded from the weather stations. The Hargreaves equation (Hargreaves 1994) was used to calculate monthly PET. SPEI is a standardized value of the climate water balance (*D*_*i*_), which is calculated as the difference between total precipitation ($P_{i}$) and ${\mathrm{PET}}_{i}$ for a given month i (Equation 1). *D*_*i*_ can be aggregated at various time scales (*k*). For example, to calculate SPEI when the time scale is set to *k* = 3 (SPEI‐3), *D*_*i*_ values are aggregated over a 3‐month period, encompassing the two preceding months and the current month. For a detailed explanation of standardization of *D*_*i*_ and SPEI calculation, see Vicente‐Serrano *et al*. (2010). The SPEI package available in R was used to calculate the SPEI index (Beguería & Vicente‐Serrano 2023).

(1) $$D_{i}=P_{i}-{\mathrm{PET}}_{i}$$

Droughts are generally categorized into four types: meteorological, vegetation or agricultural, hydrological, and socio‐economic droughts (Zeng *et al*. 2022). We are assessing the impact of droughts on European beech trees, which are deep‐rooted. Deep‐rooted trees can access water from deeper soils, making them less susceptible to short‐term moisture deficits (Maeght *et al*. 2013). When droughts are prolonged (water deficits >3–6 months), they can eventually deplete the water in deep soils used for root water uptake, leading to vegetation or agricultural droughts (Somasundaram *et al*. 2024). Such soil moisture deficits then affect trees. In the root system of European beech in Grinderwald, northern Germany, roots were sampled to a depth of 2 m (Kirfel *et al*. 2017). This is relatively shallow compared to tropical trees (root depth >8 m); therefore, European beech cannot access the very deep soil moisture available to tropical trees (Maeght *et al*. 2013), making beech more vulnerable to seasonal droughts (West *et al*. 2022). Thus, SPEI‐3 is optimal to assess drought conditions and their impact on European beech. Shallow‐rooted plants, such as grassland and crops, exhibit stress after short‐term droughts, where SPEI‐1 is appropriate for monitoring such short‐term deficits (Hunt *et al*. 2014). The SPEI‐3 was computed to assess the association between the weather and the temporal response of the forest canopy seen through computed DpRVI deviations.

### Sentinel‐1 SAR data characteristics and preparation

The ESA S1 C‐band (5.405 GHz) SAR datasets are available globally and have been freely available since 2014 (ESA 2021). The default observation mode of S1 over land is Interferometric Wide‐swath (IW) in VV and VH polarizations, covering a swath‐width of 250 km. We obtained the Level‐1 Ground Range Detected (GRD) S1 backscatter coefficient ($\sigma^{0}$) data in decibels (dB) from the Google Earth Engine (GEE) platform (accessed at Sentinel‐1 GRD dataset) by applying the following filters:

- Metadata filter (bands: VH, VV, acquisition mode: Interferometric Wide‐swath (IW) and orbit pass: ascending).
- Temporal filter (start date: 13 October 2014, end date: 3 July 2023).
- Spatial bound filter (shapefile of the study area).

All available ascending pass time series datasets were used for this study. There were 809 ascending pass S1 images, starting from 13 October 2014 to 3 July 2023. Limiting to the ascending pass, the temporal revisit varied from 1 to 5 days in our study area with both twin S1A and S1B satellites operational. S1B was launched on 25 April 2016. With only S1A operational before this date, less dense ascending pass images were available, with temporal revisit time from 5 to 12 days. Following the S1B malfunction on 23 December 2021, the temporal revisit time dropped to 5–7 days in our study area. In a few cases, images were available in intervals of 12 and 17 days. Within our study area, the incidence angle ranges from 35.84° to 38.57° and 44.25° to 45.75° for relative orbit numbers 117 and 15, respectively.

The preprocessing steps implemented by GEE to derive the backscatter coefficient are: apply orbit file, GRD border noise removal, thermal noise removal, radiometric calibration, and terrain correction. The S1 GRD dataset is available in GEE at a spatial resolution of 10 m. After which the backscatter coefficient values were converted to a linear scale. Subsequently, a boxcar smoothing was performed using a radius of 2.5 pixels and a square kernel, that is, mean of surrounding 25 pixels, to reduce speckle noise.

### Median deviations method

**Fig. 2:**
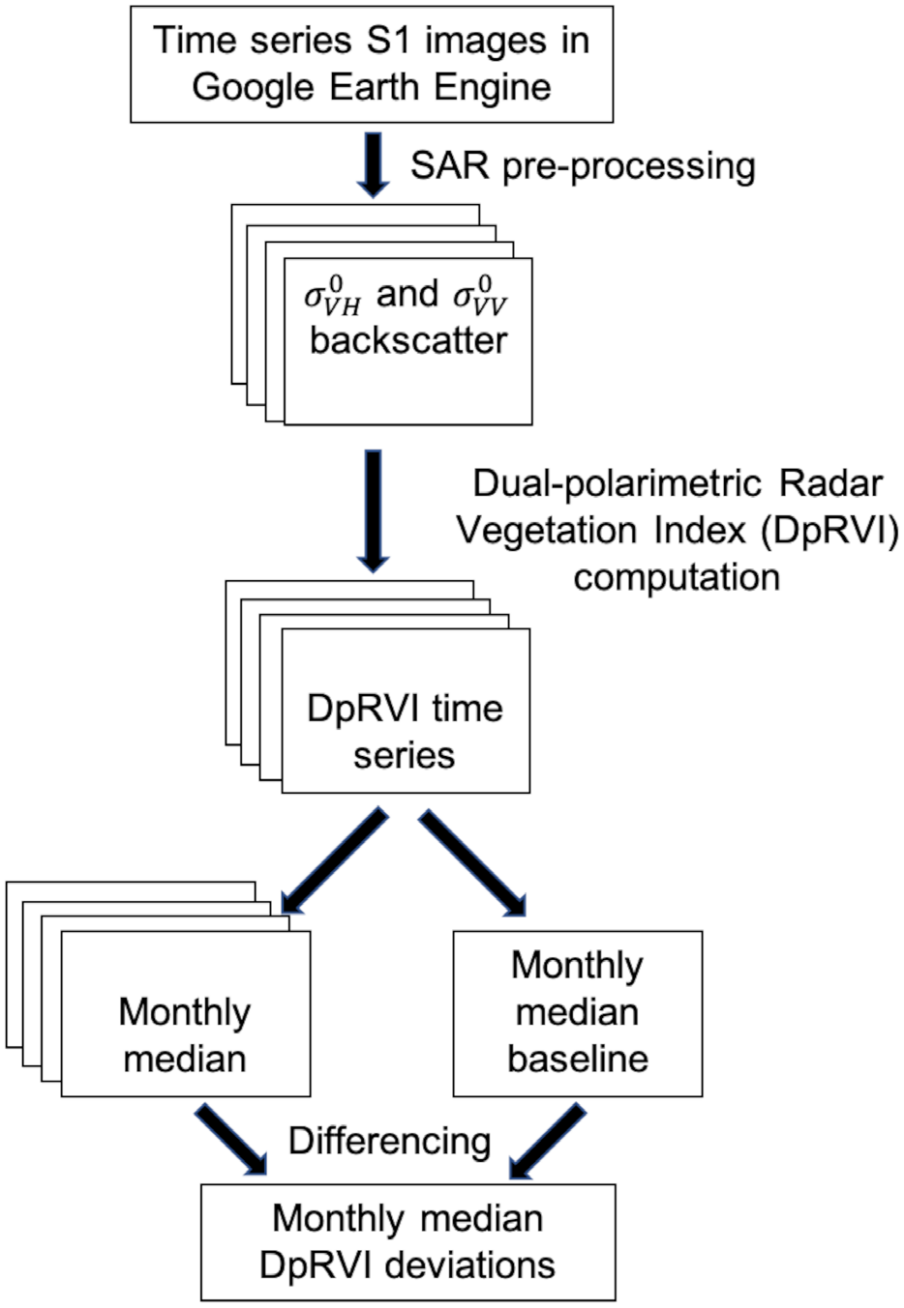
Methodological flowchart showing the process for Median Deviations calculation from dual polarimetric C‐band Sentinel‐1 data.

The methodological steps followed for calculating median deviations are presented in Fig. 2.

### Dual Polarimetric Radar Vegetation Index

The pre‐processed time series of S1 as described below, spanning 2014–2023, was used to derive the time series of the DpRVI index (Mandal, Kumar, *et al*. 2020; Bhogapurapu *et al*. 2022). Here, we follow Bhogapurapu *et al*. (2022) who proposed formulation for DpRVI using the GRD dataset instead of the Single Look Complex (SLC) used by Mandal, Bhogapurapu, *et al*. (2020), Mandal, Kumar, *et al*. (2020). Utilizing the S1 GRD SAR dataset significantly improves computational efficiency, because only the available backscatter intensities are used, instead of covariance information from the SLC dataset. This computational advantage facilitates the processing of longer time series datasets, so monitoring applications can be achieved.

The pre‐processed dual cross‐polarimetric S1 GRD dataset was used to compute the cross‐pol ratio q on a linear scale (Equation 2).

(2) $$q=\sigma_{\mathrm{VH}}^{0}/\sigma_{\mathrm{VV}}^{0}$$

Under the assumption that $\sigma_{\mathrm{VV}}^{0}\geq \sigma_{\mathrm{VH}}^{0}$ for a mono‐static antenna configuration (i.e. the transmitter and receiver are co‐located) and a natural scene, q is bounded between 0 and 1, that is, 0≤q≤1 (Cloude 2010). Then, two vegetation descriptors are computed using ratio *q*, namely the co‐pol purity parameter $m_{c}$, and the co‐pol intensity parameter $\beta_{c}$ (Equations 3 and 4, respectively) (Bhogapurapu *et al*. 2021, 2022).

(3) $$m_{c}=\frac{1-q}{1+q}\;\text{where}\;0\leq m_{c}\leq 1$$

(4) $$\beta_{c}=\frac{1}{1+q}\;\text{where}\;0\leq \beta_{c}\leq 1$$

The DpRVI is derived by subtracting the product of the co‐pol purity parameter $m_{c}$ and the co‐pol intensity parameter $\beta_{c}$ from unity (Equation 5).

(5) $$\text{DpRVI}=1-m_{c}\beta_{c}\;\text{where}\;0\leq \text{DpRVI}\leq 1$$

When the canopy is dense, the randomness and complexity of the vegetation increase, and $m_{c}$ and $\beta_{c}$ have low values, resulting in high DpRVI values. Conversely, $m_{c}$ and $\beta_{c}$ are high in a sparse canopy, which decreases the DpRVI values (Mandal, Bhogapurapu, *et al*. 2020).

The code to compute DpRVI in the GEE platform is available at: https://code.earthengine.google.com/630b7f024ac5cbae8af79b6d6c8b622f.

### Median deviations calculation

The 2014–2023 time series of the computed DpRVI index were used for computation of the baseline monthly medians and deviations from those baseline monthly medians. We chose to compute the medians from the time series, instead of the means, because the mean is affected by outliers whereas the median is robust against outliers (Leys *et al*. 2013). The time interval used for computing the median is 1 month, that is, we computed a per‐pixel monthly median DpRVI value for each year. To assess the spatial and temporal response of the forest canopy, a per‐pixel long‐term baseline DpRVI value was defined as the monthly median value across the available time series. Then, the monthly median DpRVI deviations were computed for each year as the difference between the long‐term baseline and the monthly medians.

Let $M\left(\cdot \right)$ be the median function, and let $X=\left\{X_{1}\left(i,t,y_{\text{year}}\right),X_{2}\left(i,t,y_{\text{year}}\right),\ldots ,X_{D}\left(i,t,y_{\text{year}}\right)\right\}$ be a set of D DpRVI values for pixel i in month t across all years $y_{\text{year}}=2014,2015,\ldots ,2023$. The median baseline $M_{t}\left(i\right)$ for pixel i for any month t=1,2,3,…,12 can be estimated (Equation 6).

(6) $$\begin{array}{l} \;M_{t}\left(i\right)=M(X_{1}\left(i,t,2014\right),X_{2}\left(i,t,2014\right),\ldots ,X_{D}\left(i,t,2014\right),\\ \;X_{1}\left(i,t,2015\right),\ldots ,X_{D}\left(i,t,2015\right),\ldots ,X_{D}\left(i,t,2023\right))\\ \;\text{for}\;i=1,2,3,\ldots ,N \end{array}$$

where N is the number of pixels in a DpRVI image and D is the number of images in month t.

Similarly, the median $M_{t}^{y_{0}}\left(i\right)$ for month t in a specific year $y_{0}$ is given by Equation (7), where $y_{0}$ represents an individual year selected from the set $y_{\text{year}}$, for which the median is computed separately.

(7) $$M_{t}^{y_{0}}\left(i\right)=M\left(X_{1}\left(i,t,y_{0}\right),X_{2}\left(i,t,y_{0}\right),\ldots ,X_{D}\left(i,t,y_{0}\right)\right)$$

where $y_{0}\in \left\{2014,2015,\ldots ,2023\right\}.$ Finally, the deviation $\delta_{t}^{y_{0}}\left(i\right)$ for a given month t in year $y_{0}$ is obtained as the difference between the baseline monthly median $M_{t}\left(i\right)$ and the monthly median $M_{t}^{y_{0}}\left(i\right)$ (Equation 8).

(8) $$\delta_{t}^{y_{0}}\left(i\right)=M_{t}^{y_{0}}\left(i\right)-M_{t}\left(i\right)$$

If $M_{t}^{y_{0}}\left(i\right)>M_{t}\left(i\right)$, then $\delta_{t}^{y_{0}}\left(i\right)$ is positive, which means that the canopy is not suffering more moisture stress than in baseline condition, while negative values indicate a more severe suffering.

Several studies on satellite image time series have performed anomaly detection on the detrended data (e.g. Zhou & Tang 2016; West *et al*. 2022). Therefore, the non‐parametric Mann‐Kendall Trend test (Mann 1945) was performed but no significant trend in the monthly median DpRVI time series was found, so trend removal was not necessary.

After the computation of the monthly median DpRVI and the monthly median DpRVI deviations for the entire study area, we strategically chose sample points to analyse monthly median DpRVI and monthly median DpRVI deviations over forests of different crown sizes – large or small –, and canopy density –dense or sparse. Crown sizes and densities can indicate different tree species at different development stages. First, the sample polygons chosen were well‐distributed within the study area by visually interpreting high‐resolution optical image from Maxar at 0.5 m spatial resolution for the following four categories: 1. dense canopy and big crowns, 2. dense canopy and small crowns, 3. sparse canopy and big crowns, and 4. Sparse canopy and small crowns. Then, a few pixels from each category were selected for detailed analysis of the time series, namely six points for Category 1, two for Category 2, three for Category 3, and seven for Category 4. The locations of the polygons and selected points in Categories 1–4 can be found in Figs S1– S5. To examine temporal variations, we plotted the time series of the selected pixels from the selected polygons corresponding to the four categories. We did not focus on the size of the polygons; rather, our priority was selecting pixels that corresponded to the four defined categories.

Previous studies have shown that rainfall prior to the satellite overpass can cause an abrupt increase in backscatter values (Khabbazan *et al*. 2019). To mitigate the influence of such outliers in the DpRVI time series, we applied a data trimming approach by removing the largest and smallest 5% of DpRVI values. We then calculated median deviations using the trimmed DpRVI data.

## RESULTS

### SPEI and climate data plot

**Fig. 3:**
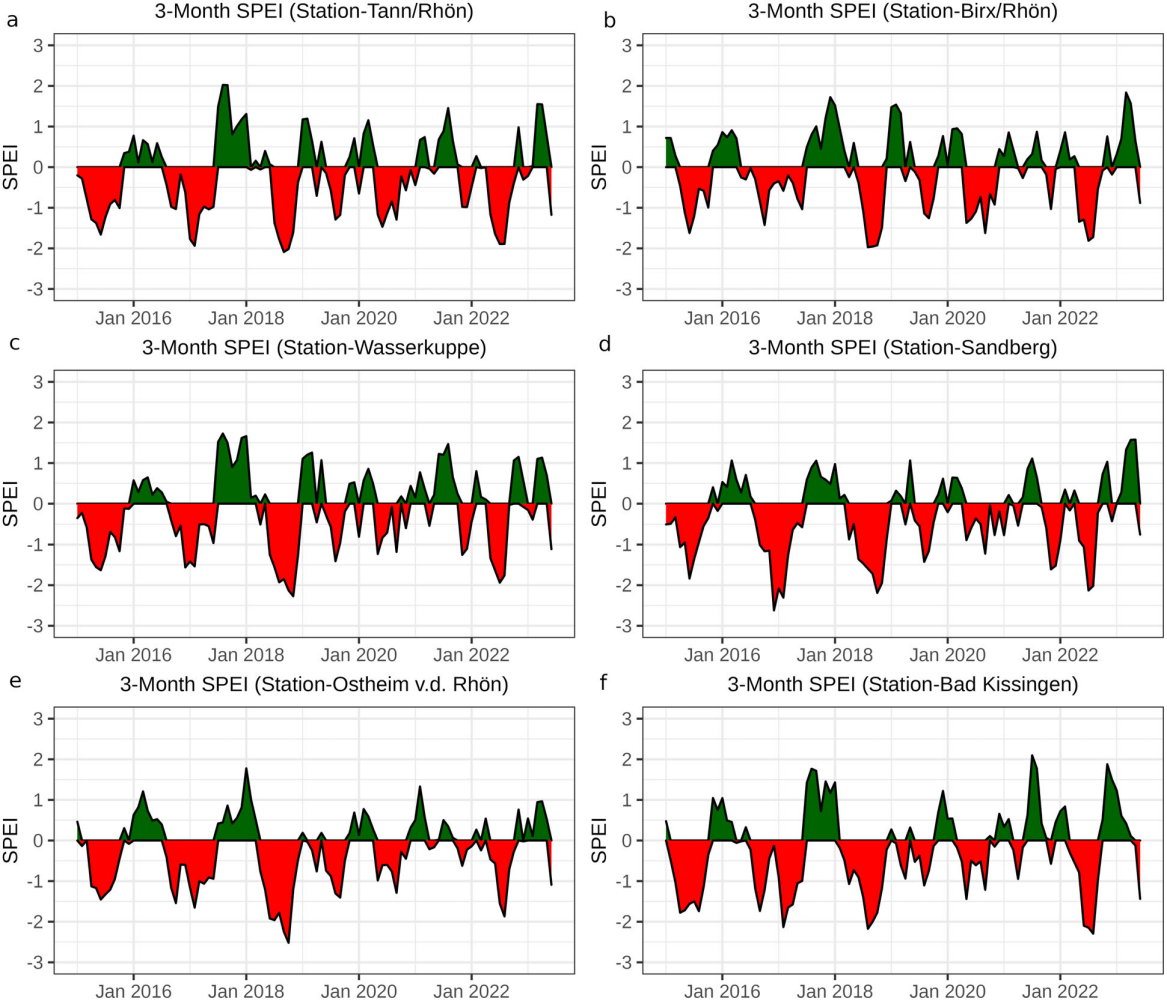
SPEI‐3 between 2015 and 2023 from (a) Tann/Rhön, (b) Birx/Rhön, (c) Wasserkuppe, (d) Sandberg, (e) Ostheim v. d. Rhön, and (f) Bad Kissingen weather stations. Negative values of SPEI (red) indicate water shortage and positive values of SPEI (green) indicate sufficient water for vegetation growth.

The 3‐month SPEI (SPEI‐3) for the weather stations are shown in Fig. 3. We chose the SPEI‐3 plot from 2015 to 2022 to align with the time period of the S1 images acquired for our study area. Positive values of SPEI signify wet conditions while negative values indicate dry conditions. A SPEI value equal to −1 is a threshold indicating drought, while SPEI values <−2 indicate extremely dry conditions (Tirivarombo *et al*. 2018). The severe droughts in 2018 and 2019 are apparent (Fig. 3).

Notably low SPEI‐3 values are observed starting in June 2018. This drought is related to reduced precipitation together with high temperatures (see also Fig. S6). During this 2018 drought, SPEI‐3 fell below −1 in June and remained below −1.5 until November. The following year (2019) was also a drought year, where SPEI‐3 fell below −1 in July. Figure 3 shows that the 2018 drought was more severe than the 2019 drought. While very low SPEI‐3 values occurred in 2016/2017, this drought event was brief and confined to December 2016–February 2017. Furthermore, this drought fell outside the growing season and thus had no severe effects. In contrast, the 2018 drought was severe and prolonged during the growing season, which made it highly damaging.

### Monthly median DpRVI time series

**Fig. 4:**
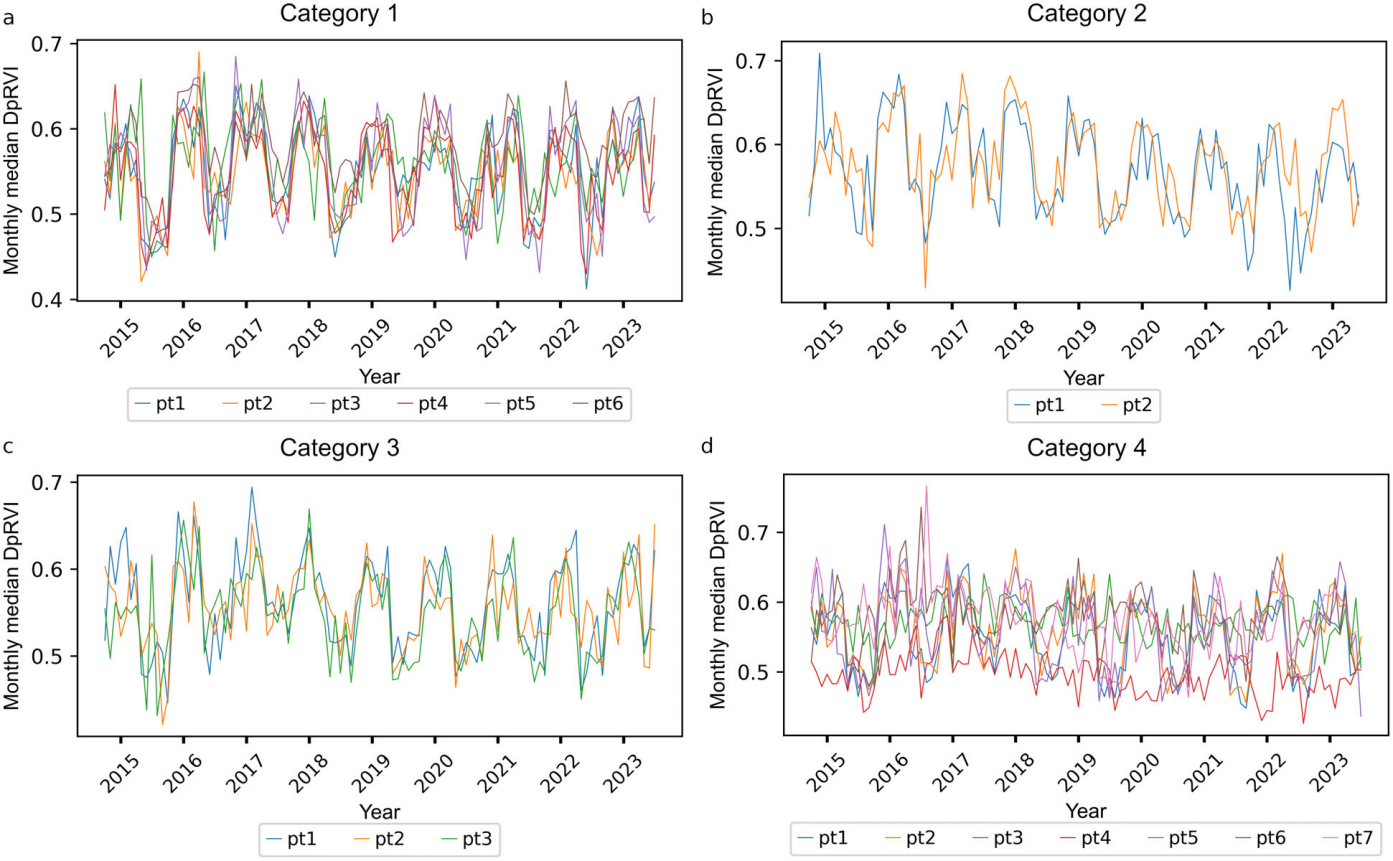
Monthly median DpRVI over all years for (a) forest with dense canopy and big crowns (Category 1), (b) forest with dense canopy and small crowns (Category 2), (c) forest with sparse canopy and big crowns (Category 3), and (d) forest with sparse canopy and small crowns (Category 4). Pt stands for point.

The monthly median DpRVI for the chosen points over the entire time series are plotted for Categories 1–4 (Fig. 4). Each time series represents data from a single pixel. The figures shown here are representative, and the same seasonal patterns were also observed in other pixels. Figure 4 shows the monthly median DpRVI for dense canopy and big crowns (Category 1) since mid 2014, where different colours indicate six different points, as described above. There is a clear seasonality, with high values (~0.6) in winter and low values (~0.45) in summer. Part of the seasonal variation in DpRVI can be explained from the underlying seasonal patterns in VV and VH backscatter related to forest phenology. As can be seen from Equation (5), DpRVI increases when $\sigma_{\mathrm{VH}}^{0}$ increases relative to $\sigma_{\mathrm{VV}}^{0}$.

Studies have characterized the seasonal phenological cycle in temperate deciduous broadleaf forest using C‐band S1 time series data (Dostálová *et al*. 2018, 2021; Frison *et al*. 2018; Rüetschi *et al*. 2018; Soudani *et al*. 2021). According to Dostálová *et al*. (2018), for broadleaf forest, VV backscatter is relatively constant throughout the year, with a slight increase in spring, while VH backscatter show a drop in spring and an increase in autumn of 0.5–2 dB. Rüetschi *et al*. (2018) focused specifically on beech and reported the same seasonal patterns.

C‐band signals are sensitive to branches and leaves of the upper forest canopy. A potential reason for the decrease in C‐band VH backscatter during spring and summer is foliage development, with two key effects: (a) signal attenuation: leaves attenuate the signals, leading to lower backscatter, (b) scatterer substitution: as leaves develop, they become dominant scatterers, replacing branches which are more reflective at C‐band. The denser the canopy, the lower the contribution of branches to backscatter. The backscatter increases again with leaf senescence and fall in autumn (Dostálová *et al*. 2018; Rüetschi *et al*. 2018; Soudani *et al*. 2021). VH polarization has higher sensitivity to volume scattering compared to VV polarization. Volume scattering is predominant in the presence of random and complex scatterers. Emergence of leaves makes the arrangement of scatterers more complex; resulting in a larger effect in VH polarization than in VV polarization (Bush *et al*. 1976; Rüetschi *et al*. 2018).

For broadleaf forest, the relatively stable VV backscatter and the VH backscatter, with a drop in spring and an increase in autumn, lead to an expected increase of DpRVI in spring and a decrease in autumn. It cannot explain the very high DpRVI values in winter, which are attributed to snow cover, acting as a volume scatterer. Snow cover over vegetation affects the SAR signa: backscatter is no longer representative of the vegetation when covered by snow (Vreugdenhil *et al*. 2020). There is snowfall throughout winter months in our study area, which coincides with high DpRVI values (see Fig. S7 for details). Given that droughts during the growing season are the largest inhibitors of tree growth and overall ecosystem productivity (Lobo‐do‐Vale *et al*. 2019), we focus on the growing season (June–September) in our detailed analysis below.

Next, we considered the monthly median DpRVI of dense canopy and small crowns (Category 2) in the same period, the monthly median DpRVI of sparse canopy and big crowns (Category 3), and the monthly median DpRVI of sparse canopy and small crowns (Category 4) (Fig. 4; different colours indicate different points for each combination of canopy density and crown size). Again, for Categories 2 and 3 there is clear seasonality, similar to Category 1, related to dense canopy (Category 2) or large crowns (Category 3). Although there could be some seasonality, for Category 4, there is much more noise over the monitoring period. This is as expected, as canopy density is much lower, and contributions to DpRVI also come from bare soils. Henceforth, we focus the analysis on the first three categories (excluding Category 4).

### Trimmed median deviations

We plotted boxplots to assess presence of significant outliers in the DpRVI time series, which may influence the calculated baseline median and monthly median values, subsequently impacting the deviations observed in DpRVI. There were large outliers. Rainfall just before image acquisition could be probable causes. An abrupt increase in VV and VH backscatter has been reported after intense rainfall due to vegetation wetness during the satellite overpass (Khabbazan *et al*. 2019). Most of the spikes in DpRVI values coincided with rainfall, obtained as 12‐h accumulated rainfall prior to the satellite overpass (see Fig. S8). Therefore, we attributed positive outliers to canopy wetness and removed them by implementing a data trimming approach, applying a 5% threshold of the largest and smallest DpRVI values, before calculating both the baseline median and the monthly median. Then, the baseline median and monthly median were computed using the trimmed DpRVI data. The baseline median and monthly median were subsequently used to calculate monthly DpRVI deviations. We did not use any time series smoothing techniques to the trimmed DpRVI data because we wanted to avoid potential loss of information in the time series and avoid shifting the time series. Smoothing can omit important short‐term fluctuations and anomalies, and distort the temporal patterns, such as sharp changes, which are of interest (Wang *et al*. 2021).

**Fig. 5:**
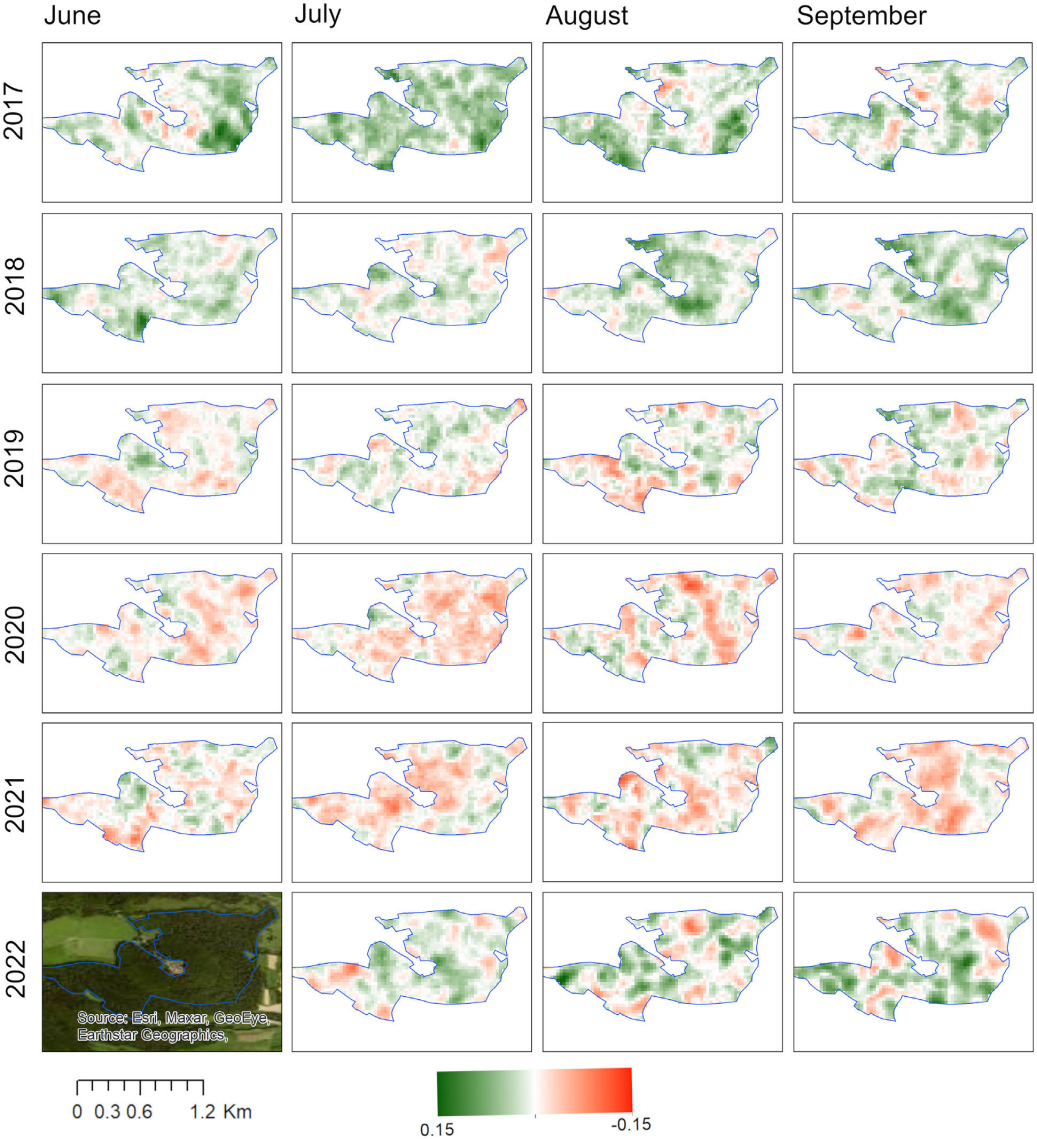
Spatial pattern of the DpRVI deviations for 2017–2022 of the broadleaf forest in a subset of the Rhön Biosphere Reserve computed from the S1 time series. The blue boundaries show the core forest areas of the Rhön Biosphere Reserve. The core forest area is shown in the optical image in the lower left corner for comparison. Monthly DpRVI anomalies are deviations from the monthly baseline, which is computed as the median DpRVI value between 2015 and 2023.

**Fig. 6:**
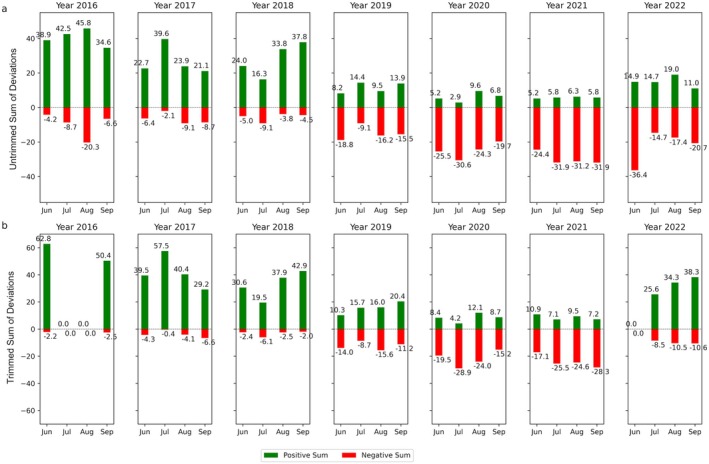
Bar graph of the sum of median deviations for the forest polygon in Fig. 5. The sum of the pixels with positive deviations (in green) and negative deviations (in red) per month from 2016 to 2022, (a) without trimming and (b) after trimming.

Since we focused on summer droughts, we selected the months June to September to plot maps of monthly DpRVI deviations for 2017–2022. The spatial pattern of DpRVI deviations between June and September of the broadleaf forest in a subset of the Rhön Biosphere Reserve is plotted in Fig. 5. Positive deviations indicate monthly median values are above the long‐term baseline of monthly median values (green in Fig. 5), whereas negative deviations indicate monthly median values are below the long‐term baseline (red in Fig. 5). We computed the sum of the pixels with positive median deviations and negative median deviations without trimming and after trimming for the forest polygon (Fig. 6).

**Fig. 7:**
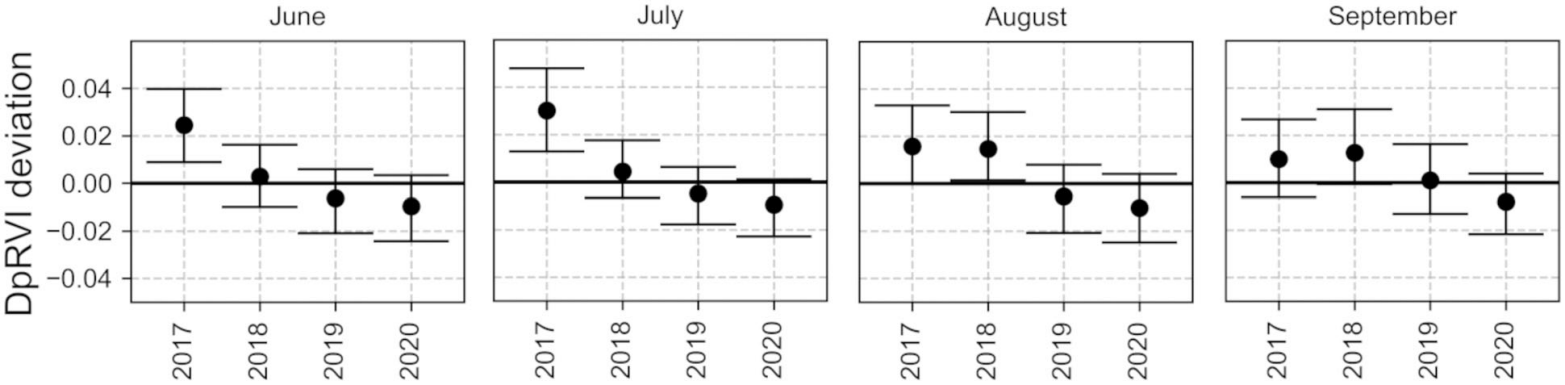
Annual DpRVI deviation (median and interquartile range) for the broadleaf forest in the Rhön Biosphere Reserve between June and September, calculateds from S1 images.

In Fig. 7, we plotted the annual DpRVI deviation for the summer months, June to September. We compared this with the annual NDVI deviation in Fig. 5 of West *et al*. (2022). This study was also performed in the broadleaf forest of the Rhön Biosphere Reserve, using NDVI as a drought indicator, which is a well‐established method for detecting and assessing droughts in forests. It is relevant to compare these results with those obtained using DpRVI to determine whether similar drought patterns in both time and space are observed. There was a very similar pattern, with small and varying deviations. The largest difference was for August. It is reasonable that what we see in DpRVI as a drought effect accumulated over the years 2018, 2019–2020. Another source of difference could be because West *et al*. (2022) used a shorter time‐period, 2017–2021, while we processed the dataset available for 2015–2023.

## DISCUSSION

The calculated SPEI captures both precipitation and temperature anomalies, enabling detection of the onset, duration, and severity of droughts in the study area. For the climate station Sandberg, substantially low precipitation and high temperatures were most pronounced during the summer growing period of 2018, persisting until the end of the year. The SPEI‐3 values exhibited a strong decline, falling below −1 in July 2018 and remaining consistently below this threshold for the remainder of the year, with the highest (<−2) observed in August (Fig. 3, Fig. S6). This decline is consistent with the forest response, as indicated by DpRVI values. Negative DpRVI deviations were observed, reflecting the onset of drought stress in July 2018, which intensified in the following years (Fig. 6b).

The summer of 2017 brought considerable rainfall that persisted until the end of the year (Fig. S6). This ensured that water was still available in 2018 (Haustein *et al*. 2023), therefore, there was less damage observed, while the onset of damage is clearly visible for July 2018, as indicated by a noticeable increase in negative DpRVI deviations, in contrast to the predominantly positive DpRVI deviations observed in July 2017 (Fig. 5). This observation aligns with the SPEI‐3 value, which fell below −1 in July 2018, confirming the onset of drought (Fig. 3).

Much like the drought in 2018, the subsequent drought in 2019 saw SPEI‐3 persist below 0 for most of the year, dropping below −1 in July (Fig. 3, Fig. S6). However, the severity of the drought in 2018 was greater than that in 2019. Forest in 2018 started to show stress which accumulated in the subsequent droughts of 2019 and 2020, as is exhibited by larger negative sums (Fig. 6).

In 2021, there was an increase in precipitation compared to 2018 and 2019, making it an average year (Fig. 3, Fig. S6). Despite overall average conditions in 2021, damage to the forest was still high (Fig. 5), suggesting that the forest had not fully recovered from the cumulative droughts of 2018–2020. Haustein *et al*. (2023) showed that forests require several years to recover from droughts, supporting our interpretation that the legacy effects of these droughts continued to impact forest health.

In 2022, severe drought conditions returned, as indicated by SPEI‐3 values of −2 for all stations (Fig. 3). Consequently, the forest exhibited renewed drought impacts (Fig. 5). However, subtle signs of recovery were also observed in 2022, as demonstrated by a slight decrease in magnitude of negative sums (Fig. 6) and a reduction in negative DpRVI deviations (Fig. 5). This recovery may be related to the water made available from the previous year's rainfall in 2021.

Anderegg *et al*. (2020) found that in the event of consecutive droughts, the subsequent droughts had greater detrimental effects compared to the initial droughts, and the vulnerability of forests increases with multiple droughts, as opposed to single drought occurrences. We also discovered that the impacts of forest droughts were intensified by the sequential occurrence of summer droughts from 2018 to 2020 in our study area. Even though deciduous trees often have the potential to recover from droughts, the extensive droughts of 2018–2020 had a strong negative impact on crown conditions and therefore, tree canopy recovery was delayed. In our study area, a plausible explanation for the observed decline in DpRVI values during droughts is a decrease in canopy moisture content. This aligns with findings of Pirotti *et al*. (2023), who reported that S1 backscatter intensity decreases in temperate broadleaf forests that have a decrease in canopy moisture content. We lack in situ measurements of canopy moisture content to confirm this directly, and we emphasize the need for validation using in situ data.

For the Rhön forest, there are no weather stations in the study area, but several stations in the surrounding area. These stations had similar SPEI patterns and could be used to estimate SPEI and drought episodes for the study area, which in turn could be related to decreases in DpRVI. Precipitation and temperature can be highly variable over an area and are influenced by topography. When no weather data are available over an area, DpRVI variations cannot be uniquely attributed to drought, but could also be related to pests and diseases. Similarly, harvested areas should be excluded from the study. A longer period of data, typically more than 30 years, is used in most studies to define a long‐term baseline for anomaly calculation. However, our study uses all S1 images available for our study area at the time of analysis (start date: 13 October 2014, end date: 3 July 2023). The use of a relatively short time‐period considering multiple years of drought occurrences from 2016 to 2023 might have affected the robustness of the long‐term baseline and the DpRVI deviation calculation. Future research might be improved by incorporating a longer time series of S1 images as additional data become available.

The application of the present DpRVI‐based methodology to detect drought effects on forests could be applied to other dense broadleaf forests, without adaptation. As shown by forest Category 4, with sparse and small tree crowns, it is not suitable for open forests, where the backscatter is not mainly dependent on the canopy but also largely on the soil and the low vegetation. This study uses a straightforward statistical method that can be easily implemented at a regional scale, without the requirement of complex processing and physical inversion.

## CONCLUSION

In this study, we applied S1‐derived DpRVI to assess long‐term drought effects in the broadleaf forest canopy for the first time. The analysis of the chosen S1 index reveals the forest response to drought. Through the free availability of longer time series of S1 images globally, coupled with the straightforward computation process of the DpRVI deviations, it is possible to employ S1‐derived DpRVI for investigating drought effects across large forest areas. Further study is required to examine which part of DpRVI decline can be attributed to decline in canopy moisture content and which part to changes in the forest structure.

## AUTHOR CONTRIBUTIONS

Conceptualization, BR and WB; methodology, BR; software, BR; validation, BR; formal analysis, BR and WB; data curation, BR; writing—original draft preparation, BR; writing—review and editing, BR, WB, HA and AS; visualization, BR; supervision, WB, HA and AS. All authors have read and agreed to the published version of the manuscript.

## Supporting information


**Fig. S1.** The selected forest polygons in the four categories. Categories: 1. dense canopy and big crowns, 2. dense canopy and small crowns, 3. sparse canopy and big crowns, and 4. Sparse canopy and small crowns. The zoomed‐in views of the selected polygons for each category are in Figs S2– S5 respectively.

**Fig. S2.** Chosen Category 1‐dense canopy and big crown sample points shown using red circles.

**Fig. S3.** Chosen Category 2‐dense canopy and small crown sample points shown using yellow circles.

**Fig. S4.** Chosen Category 3‐sparse canopy and big crowns sample points shown using purple circles.

**Fig. S5.** Chosen Category 4‐sparse canopy and small crowns sample points shown using blue circles.

**Fig. S6.** SPEI and climate data plot of the climate station Sandberg. (a) SPEI‐3 for the years 2015–2022. (b) Monthly mean temperature and monthly total precipitation.

**Fig. S7.** The daily station observations of snow depth in cm plotted as blue bars for weather station Wasserkuppe from 2015 to 2023. Monthly median DpRVI from 2015 to 2023 of the selected six points of type dense canopy and big crowns (Category 1) plotted in line graphs. High DpRVI values in winter coincide with snowfall and can be attributed to the volume scattering of snow.

**Fig. S8.** The DpRVI time series of the five randomly selected pixels in the forest polygon in Fig. 5 plotted along with bar showing accumulated rainfall 12‐h prior to the overpass of the satellite for climate station Sandberg. Bar graphs of median deviations.
