# Supplementary figures and images for: Dual Polarimetric Radar Vegetation Index for monitoring forest moisture stress using time series of Sentinel‐1 SAR data
Source: Plant Biol (Stuttg). 2025 May 16;28(3):978–90. doi: 10.1111/plb.70036 (PMC13089602; doi:10.1111/plb.70036)

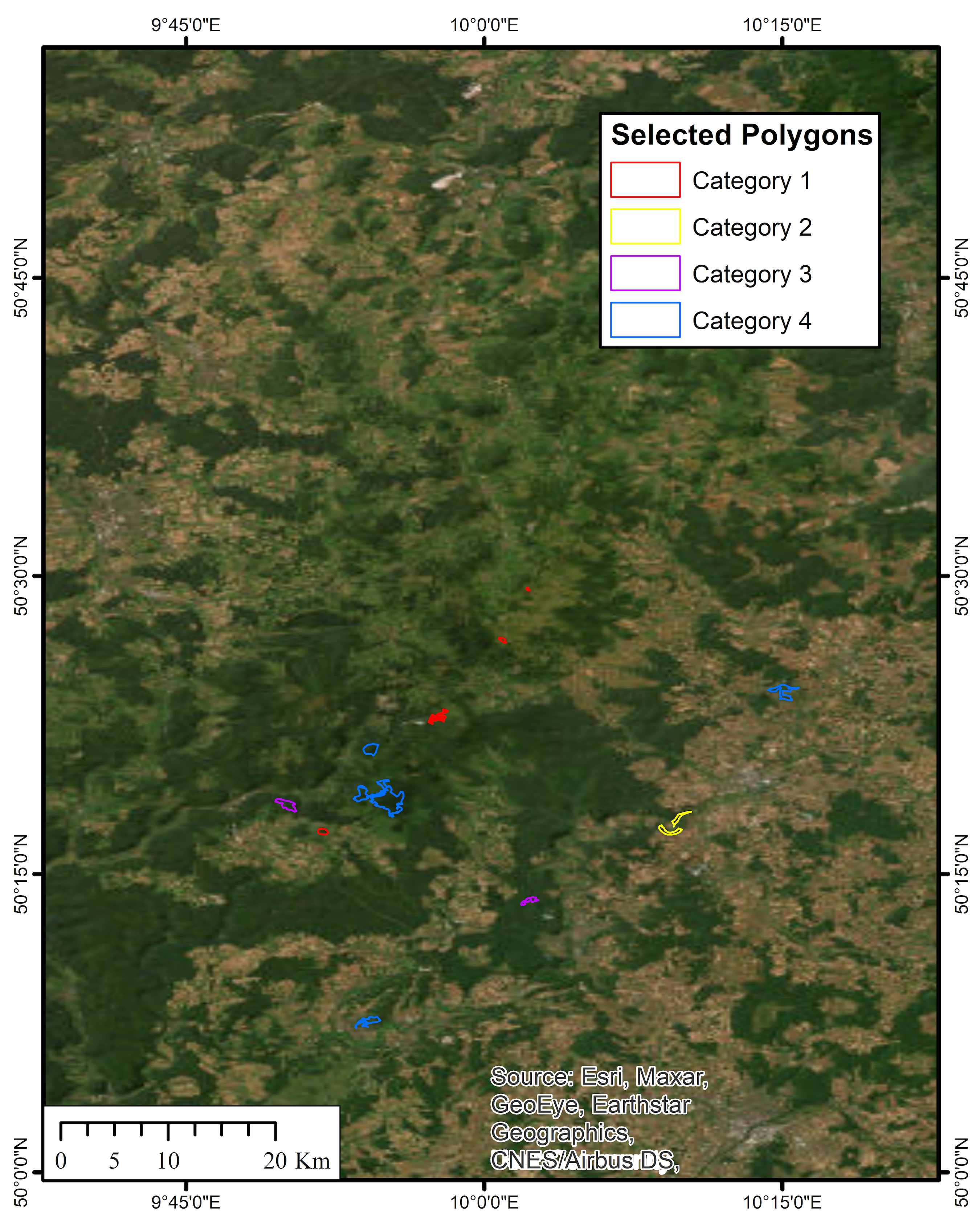

Supplement: Supplementary file 1 — Fig. S1. The selected forest polygons in the four categories. Categories: 1. dense canopy and big crowns, 2. dense canopy and small crowns, 3. sparse canopy and big crowns, and 4. Sparse canopy and small crowns. The zoomed‐in views of the selected polygons for each category are in Figs S2– S5 respectively. [file PLB-28-978-s007.tif]

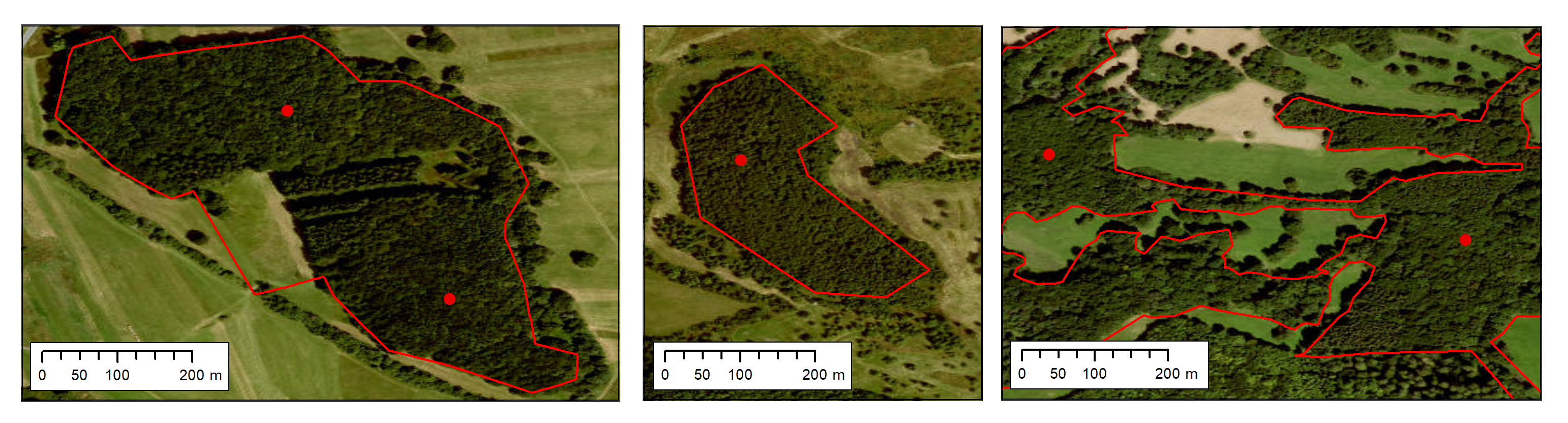

Supplement: Supplementary file 2 — Fig. S2. Chosen Category 1‐dense canopy and big crown sample points shown using red circles. [file PLB-28-978-s001.tiff]

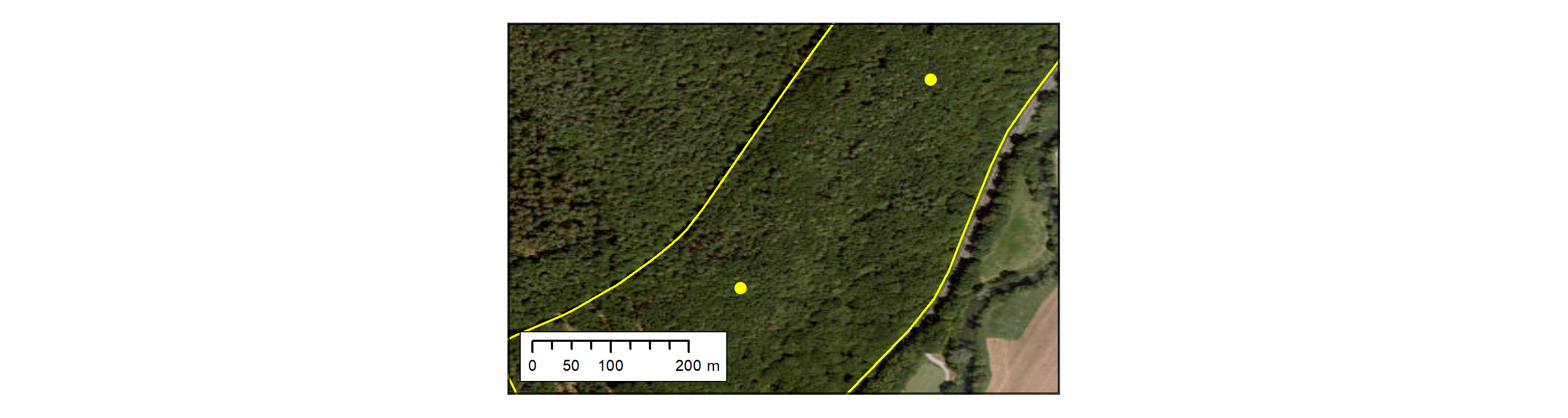

Supplement: Supplementary file 3 — Fig. S3. Chosen Category 2‐dense canopy and small crown sample points shown using yellow circles. [file PLB-28-978-s002.tif]

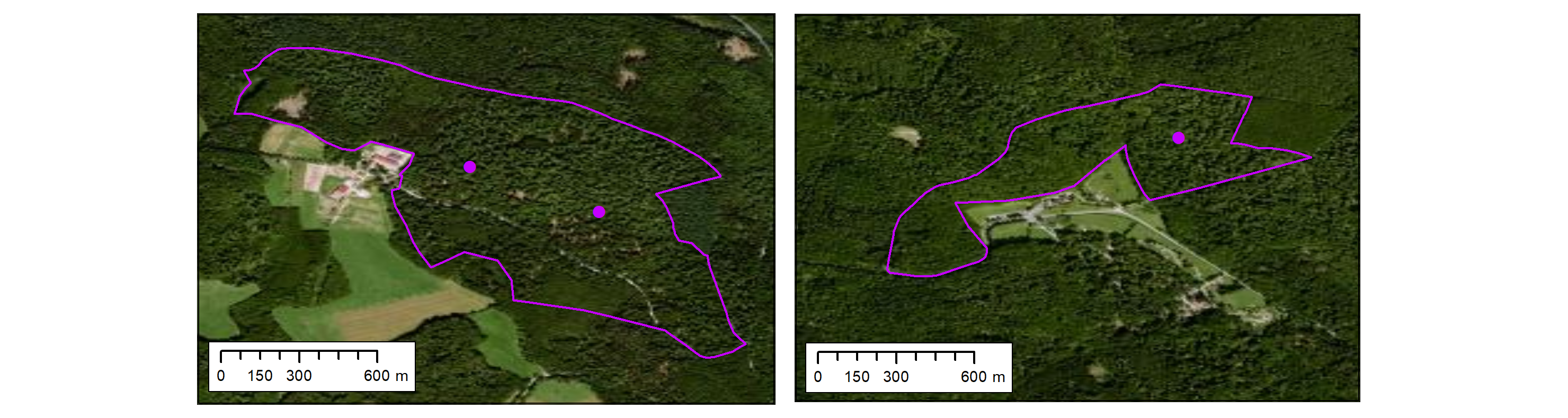

Supplement: Supplementary file 4 — Fig. S4. Chosen Category 3‐sparse canopy and big crowns sample points shown using purple circles. [file PLB-28-978-s006.tif]

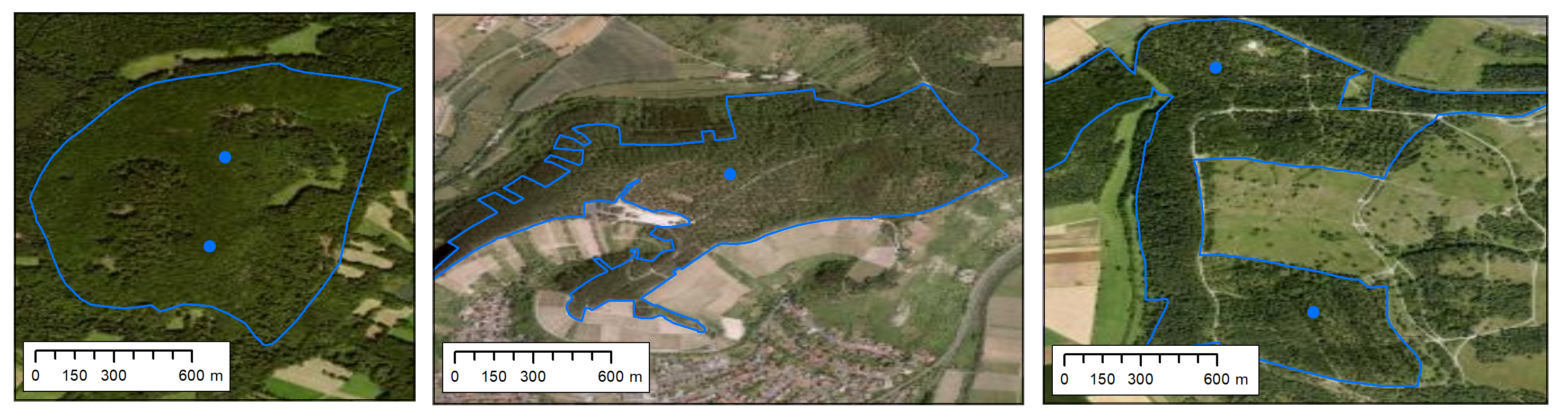

Supplement: Supplementary file 5 — Fig. S5. Chosen Category 4‐sparse canopy and small crowns sample points shown using blue circles. [file PLB-28-978-s004.tiff]

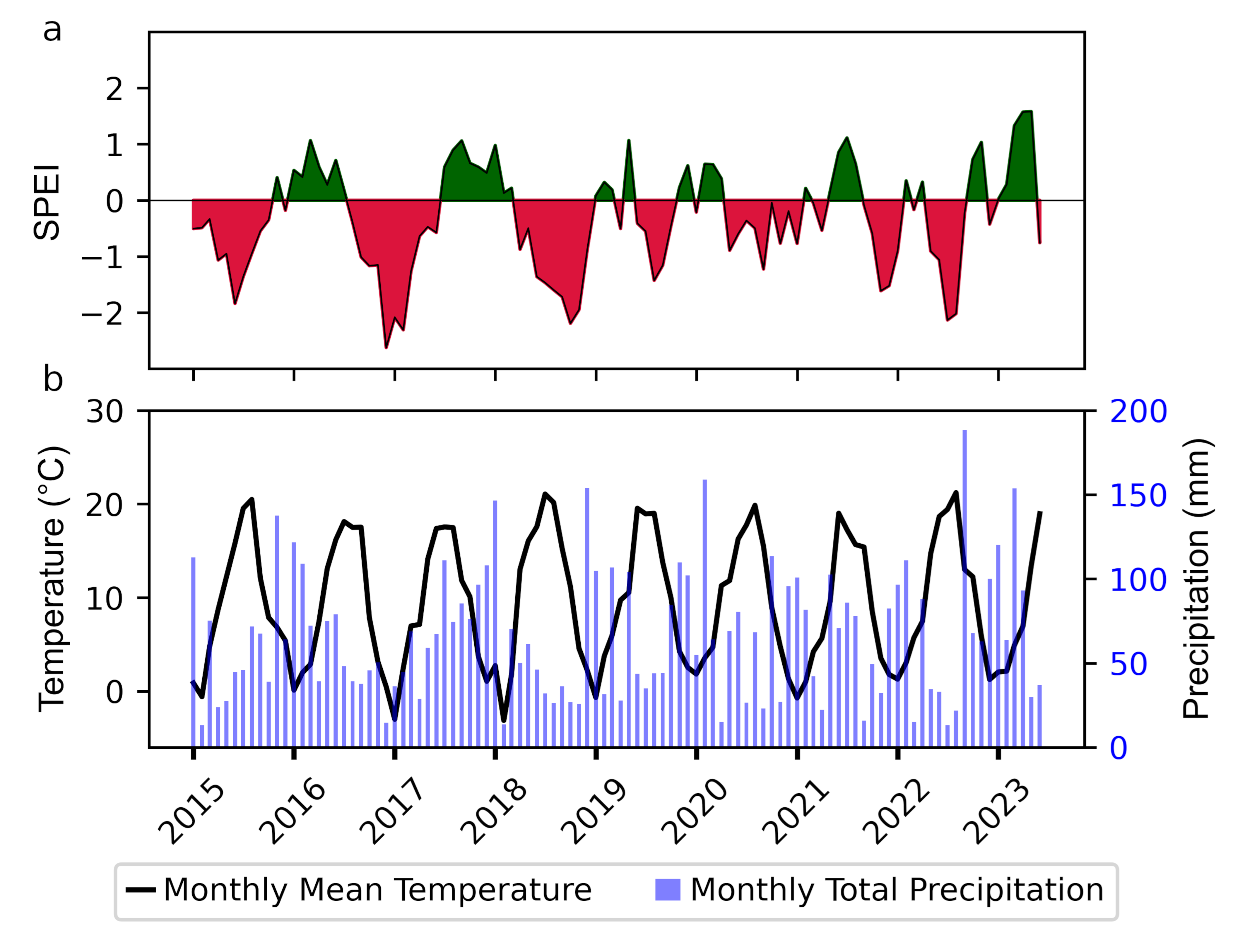

Supplement: Supplementary file 6 — Fig. S6. SPEI and climate data plot of the climate station Sandberg. (a) SPEI‐3 for the years 2015–2022. (b) Monthly mean temperature and monthly total precipitation. [file PLB-28-978-s008.tif]

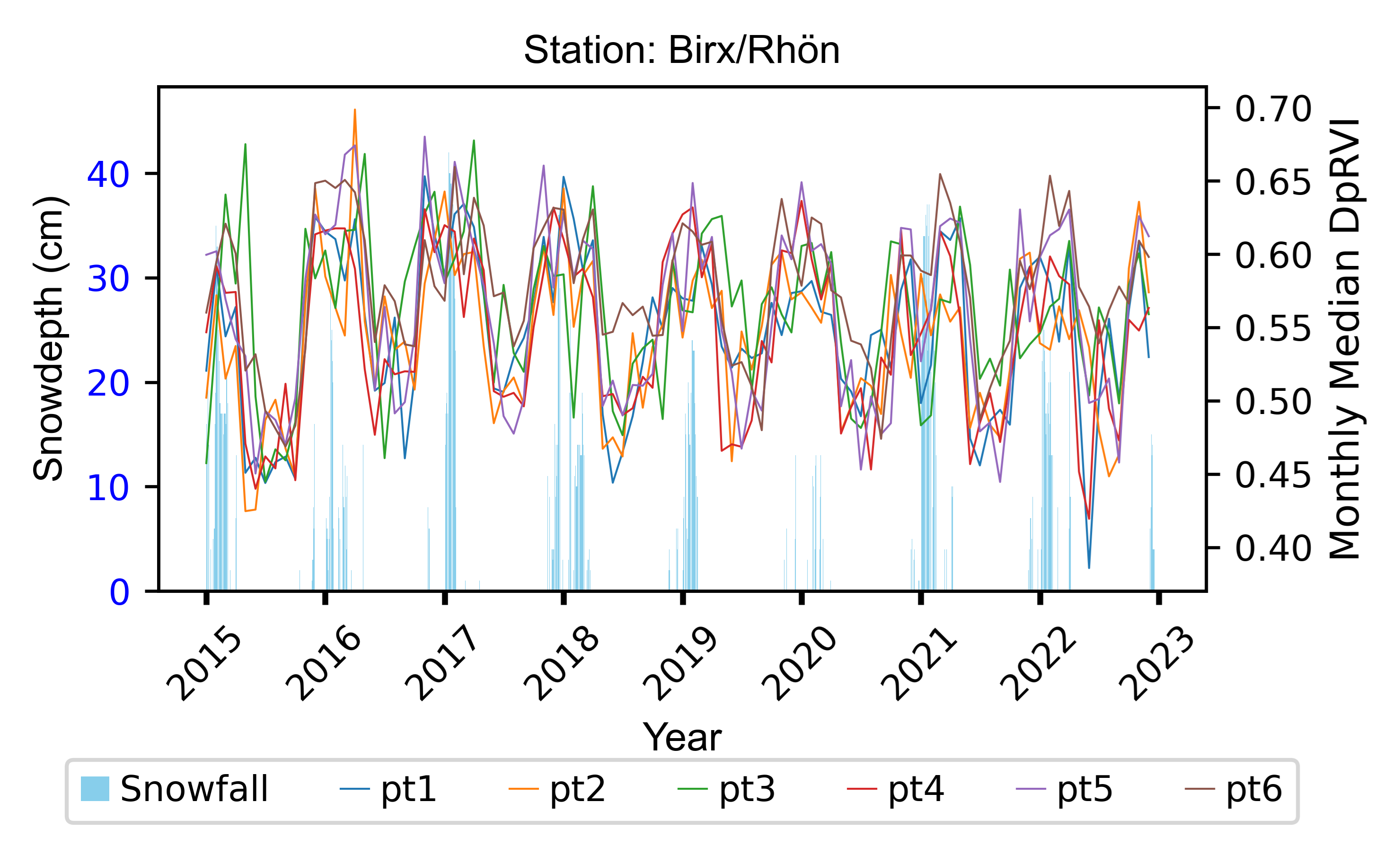

Supplement: Supplementary file 7 — Fig. S7. The daily station observations of snow depth in cm plotted as blue bars for weather station Wasserkuppe from 2015 to 2023. Monthly median DpRVI from 2015 to 2023 of the selected six points of type dense canopy and big crowns (Category 1) plotted in line graphs. High DpRVI values in winter coincide with snowfall and can be attributed to the volume scattering of snow. [file PLB-28-978-s005.png]

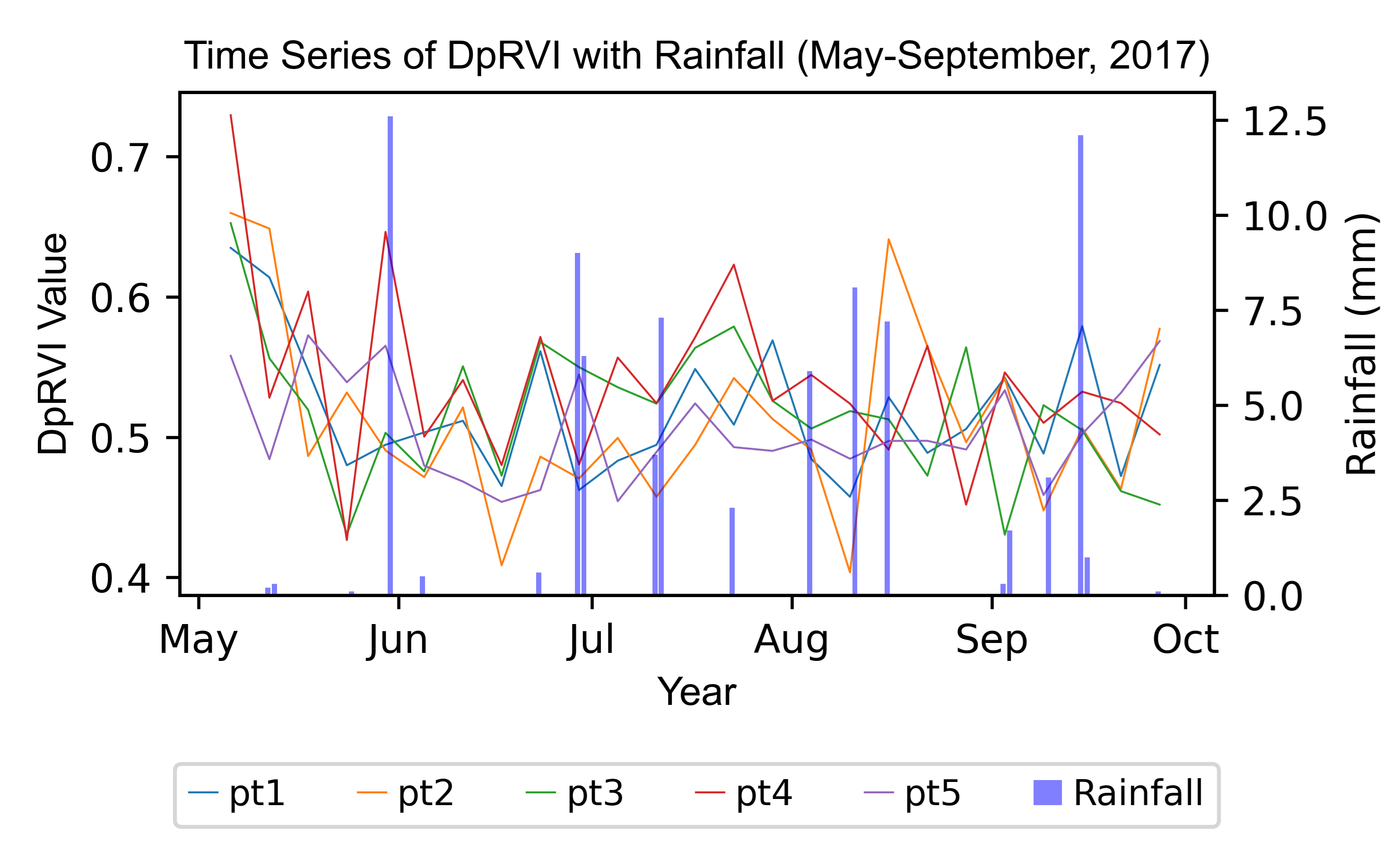

Supplement: Supplementary file 8 — Fig. S8. The DpRVI time series of the five randomly selected pixels in the forest polygon in Fig. 5 plotted along with bar showing accumulated rainfall 12‐h prior to the overpass of the satellite for climate station Sandberg. Bar graphs of median deviations. [file PLB-28-978-s003.png]
